# Acceptability and Concordance of Self- Versus Clinician-Sampling for HPV Testing among Rural South Indian Women

**DOI:** 10.31557/APJCP.2021.22.3.971

**Published:** 2021-03

**Authors:** Purnima Madhivanan, Holly Nishimura, Kavitha Ravi, Benjamin Pope, Makella Coudray, Anjali Arun, Karl Krupp, Poornima Jaykrishna, Vijaya Srinivas

**Affiliations:** 1 *Department of Health Promotion Sciences, Mel & Enid Zuckerman College of Public Health, University of Arizona, Tucson, USA. *; 2 *Public Health Research Institute of India, Mysore, India. *; 3 *Departments of Medicine, Family & Community Medicine, College of Medicine, University of Arizona, Tucson, USA. *; 4 *Department of International Health, Bloomberg School of Public Health, Johns Hopkins University, Baltimore, USA. *; 5 *Department of Epidemiology & Biostatistics, Mel & Enid Zuckerman College of Public Health, University of Arizona, Tucson, USA. *; 6 *Department of Population Health Sciences, College of Medicine, University of Central Florida, Orlando, USA. *

**Keywords:** Screening cervical cancer, self-collection, self-sampling, India

## Abstract

**Background:**

Despite being largely preventable, HPV-related cervical cancers continues to be the second highest cause of cancer deaths among Indian women. HPV testing using self-sampled samples may offer an opportunity to expand cervical cancer screening in India where there is currently a shortage of providers and facilities for cervical cancer screening. The study examines acceptability and concordance of self vs. clinician collected samples for HPV-relted cervical cancer screening among rural South Indian women.

**Methods:**

Between May and August 2017, eight mobile screening clinics were conducted among 120 eligible women in rural villages in Mysore District, India. Women over the age of 30 underwent informed consent process and then self-sampled a sample for cervicovaginal HPV DNA testing. Next, the women underwent clinical exam where the clinician collected a cervicovaginal HPV DNA sample. Following the clinical exam, all participants answered an interviewer-administered questionnaire to assess their history of cervical cancer screening and acceptability of self- and clinician-sampling methods. To assess diagnostic accuracy, concordance of self- and clinician-sampled HPV DNA specimens was calculated in addition to five measures of acceptability (feeling of caring, privacy, embarrassment, genital discomfort, and genital pain).

**Results:**

Study participants had a median age 39 years, about four-in-ten (41.7%) had a secondary education or above, the vast majority (87.5%) were married and only 3.4% reported having screened for cervical cancer. For all measures of participant acceptability, self-sampling was rated significantly higher than clinician-sampling. Cohen’s kappa was 0.73 (95% CI: 0.34, 1.00), indicating substantial agreement between self- and clinician-sampling.

**Conclusion:**

This study demonstrates that HPV self-sampling for cervical cancer screening is feasible and acceptable in a community setting among South Indian rural women. Concordance between self-sampling and clinician-sampling was adequate for screening in community settings.

## Introduction

Cervical cancer is the fourth most common cancer in women worldwide, and the seventh overall, with an estimated 570,000 incident cases per year with 311,000 women dying from the disease (Ferlay et al., 2015; World Health Organization, 2020). Approximately 84% of cases and 88% of cervical cancer deaths occur in low- and middle-income countries (Randall and Ghebre, 2016; Arbyn et al., 2020). High income countries have been effective in reducing cervical cancer incidence and mortality due to population-based cytologic screening programs (Kamangar et al., 2006; Ferlay et al., 2015; Beddoe, 2019). Cytology-based screening, conducted as part of a pelvic examination, is a method of detecting pre-invasive neoplasia (also known as pre-cancerous lesions). Detection and treatment of neoplasia at the pre-invasive stage can prevent lesions from becoming cancerous. Advances in cytology-based screening, however, have not translated to less developed regions including countries in Africa, South and Southeast Asia, and parts of Latin America where cervical cancer is the second most common cancer. 

To address many of the barriers to cervical cancer screening, the World Health Organization recommends low-cost, culturally-acceptable alternatives to cytology such as Human Papillomavirus (HPV) DNA testing (WHO). Persistent HPV infection is associated with the vast majority of cervical cancer cases (WHO). Tests for HPV DNA are easily reproducible and have higher sensitivity for detecting high-grade cervical intraepithelial neoplasia than cytological tests (WHO; Sankaranarayanan et al., 2011; Bansil et al., 2014; Sankaranarayanan, 2014; Racey and Gesink, 2015). A meta-analysis found an overall sensitivity of 80-95% for HPV testing compared to 60-80% for cytologic testing, and an overall specificity of 50-70% for HPV testing compared to 85-95% for cytologic testing (Sahasrabuddhe et al., 2012). Unlike cytologic testing, HPV testing does not require retesting or multiple visits from the patient. HPV testing also offers an option for self-sampling where cytological testing does not.

Due to the physical and psychological discomfort associated with the pelvic exam necessary for cytologic testing, self-sampling may be an acceptable alternative. Furthermore, Indian women continue to be hesitant and shy about undergoing a pelvic exam culturally. The self-sampling method addresses structural issues to resource constraints, lack of staff, and space to provide screening. A recent systematic review of 37 self-collection studies from 24 countries found overall high acceptability (Nelson et al., 2017). A self-sampling option may be appealing to women that would otherwise not screen. 

The Indian ministry of Health and Family Welfare has been piloting cervical cancer screening programs in select states since November 2016 (Basu et al., 2019; Kedar et al., 2019). India with the world’s second largest population, also experiences a large proportion (27%) of the world’s cervical cancer deaths annually (Sankaranarayanan, 2014). Each year 96,922 women are diagnosed with cervical cancer and 60,078 die from the disease (Sankaranarayanan et al., 2011; National Institute of Cancer Prevention and Research and Indian Council of Medical Research, 2018). The screening program required mandatory oral, breast, and cervical cancer screening for people over the age of 30 in 100 selected districts of India (Bagcchi, 2016). While preliminary data regarding the overall impact of cancer screening program is not available, three hospital based studies showed promising results (Sowjanya et al., 2005; Mehtal, 2017; Kuriakose et al., 2020). If the nation-wide screening program is to be successful in the culturally and economically diverse regions of India, feasibility and acceptability of different screening methods must be identified and addressed.

This study was conducted in a community-based setting in rural communities in the state of Karnataka among asymptomatic women attending a mobile cervical cancer screening program. The objective was to assess the feasibility and acceptability of self-sampling for HPV testing as compared to clinician-collected sample. 

## Materials and Methods

*Study Sample/Population*

This was a cross-sectional study that collected data between May and August 2018. Two-hundred participants were recruited in order to obtain a sample size of 120 as a convenience sample from the community. Inclusion criteria for participation was being 30 years or older, who have not undergone cervical cancer screening within the last three years, and having the capacity to undergo informed consent process. Women who had had a hysterectomy, currently menstruating, pregnant or had been diagnosed with cervical cancer were excluded from the study. The women who were menstruating were invited to enroll after menstruation.

*Ethical approvals*

Only women who were able to give informed consent were recruited to participate in the study. The study research protocol was reviewed and approved by the institutional ethics committees of Public Health Research Institute of India and University of California, Berkeley. 

*Recruitment*

The study was conducted at the Public Health Research Institute of India (PHRII) mobile cervical cancer screening program in Mysore, Karnataka, India. A study staff member invited women in the waiting area awaiting a pelvic exam as part of the standard of care for cervical cancer screening, to participate in the study. The mobile clinics were set up in eight rural villages of Mysore District in cooperation with District surveillance officer who represents the District Health and Family Welfare Officer, Mysore.

*Screening*

Women who were eligible to participate were seen by a member of the research staff to obtain informed consent in Kannada, the predominant language spoken in the region. Participants then answered an interviewer administered brief questionnaire to collect socio-demographic, reproductive, and health data, as well as an assessment of knowledge regarding cervical cancer. After the screening was completed, they completed a short interviewer-administered survey on acceptability of the collection method. 

*Procedure*

After the screening questionnaire was completed by the study staff member, the participant was asked to self-collect a vaginal swab in the privacy of their homes or in a private space near the mobile clinic. Women were given verbal and pictorial instructions prior to sample-collection about the use of a cervical brush, which was then swirled and stored in the PreservCyt Solution. Briefly, the woman was instructed to insert the swab two inches into her vagina, swirl three times along the upper vaginal wall, then to remove and place the swab into the provided collection tube and return to the research staff. Swab insertion and removal was not witnessed by the research staff member. The cervicovaginal sample was collected using a broom-type collection device (Digene HC2 NA Collection Device) and then placed in a 1ml of specimen transport medium (STM) containing PreservCyt solution to prevent drying of the sample. At the time of receipt, the study interviewer verified if the swab was completely submerged into the solution and the collection bottle was closed correctly.

As part of the standard of care for cervical cancer screening, a clinician performed a pelvic examination. Lubricant was not applied to the speculum prior to insertion. After speculum placement, the cervical os was visualized and a cotton-tipped swab was used to remove any excess secretions. An endocervical specimen was collected by inserting the brush-like collection device into the cervical os and rotating five times. This was placed in the collection device which was then sent to PHRII laboratory by maintaining cold/refrigerated condition for further testing for HPV. After the completion of the examination, the research interviewer administered an acceptability questionnaire about the woman’s experiences with the pelvic exam and self-sampling processes. The questionnaire was a Likert scale, that ranged from 1(least favorable) to 5(most favorable), to evaluate experiences related to perception of care, comfort, privacy, embarrassment, and pain for both collection methods individually. Measures used in the acceptability questionnaire have been previously used in other HPV self-sampling studies. The questionnaire also assessed difficulties encountered during self-sampling and questions regarding the ability to perform the self-sampling.

*Testing*

The Digene HC2 NA Collection Device was used for self- and clinician-sampling. Both samples were transported to and tested at the PHRII laboratory using the digene Hybrid Capture 2 HPV DNA Test, on a Qiagen platform which detects 13 high-risk HPV types (16/18/31/33/35/39/45/51/52/56/58/59/68). Results were reported as positive or negative for high and low risk types. 

*Data Analysis*

Summary statistics were generated for demographic variables and clinical characteristics. Additionally, summary statistics were tabulated for preferences for cervical cancer screening, including gender preference of the provider, collection type preference (physician or self), whether the respondents would test with self-sampling again, and whether they would recommend self-sampling to a friend. A 2-by-2 table was generated to compare self-sampling versus clinician-sampled specimens. From this table, performance measures and their associated 95% confidence intervals were generated, including sensitivity and specificity, predictive values positive and negative, and the proportion of concordant pairs and the proportion of concordant pairs among those pairs for which the clinician-sampled sample was HPV DNA testing. Any indeterminate index test or reference standard results were treated as missing. Missing data were excluded from analyses, that is, no imputation was performed.

Acceptability measures included: how well cared for respondents felt; how well the respondents’ privacy was handled during the tests; the extent to which respondents felt embarrassed during the test; whether the test caused the respondents genital discomfort; and whether the test caused the respondents’ genital pain. 

We used the following formula to determine the sample size for the study: 

The formula suggested that a sample size of 100 would result in a conﬁdence interval 

estimated to be 0.05. This was consistent with recommendations from McAlinden et al that sample sizes for agreement studies should include at least 100 subjects(McAlinden et al., 2011). We overrecruited by 20% to account for incomplete data and ‘indeterminate’ test results.

Data were compared using a paired t-test, or, as appropriate, the Wilcoxon signed-rank test. Cohen’s kappa, a measure of agreement between two raters, was also computed. For Cohen’s kappa, a value near zero represented agreement being due to random chance, 0.01-0.20 represented slight agreement; 0.21-0.40, fair agreement; 0.41-0.60, moderate agreement; 0.61-0.80, substantial agreement; and 0.81-1.00 almost perfect agreement. All tests were carried out using an α=0.05 significance level. Stata version 11.2 (StataCorp, College Station, TX) was used for all analyses except for Cohen’s Kappa and its associated confidence interval, for which the ckap command, part of the rel package in R version 3.6.2 (R Core Team 2019), was used.

## Results

Sociodemographic characteristics of the study participants is in Table 1. Among the participants, the median age was 39 (IQR: 32-45), while the median monthly income was 5,000 INR (IQR: 3,000-10,000). The highest percentage of respondents (41.7%, n=50) had secondary or higher education, while the majority (n=113, 95.0%) were Hindu, married (87.5%, n=105), and never smokers (n=113, 94.2%). About three-fifths of respondents (57.1%) reported having never been screened for cervical cancer. 

Overall prevalence of HPV DNA positivity in the clinician sampled endocervical swab samples was 12.6% (95%CI: 7.7, 19.9).The self-sampled vaginal swab, when compared to the gold standard of the clinician-sampled endocervical swab for HPV DNA testing, had a greater specificity (98.1%; 95% CI: 95.5, 100) than it did sensitivity (66.7%; 95% CI: 42.8, 90.6). The overall agreement between the two tests was 94.1% (95% CI: 88.1, 97.3). The Cohen’s kappa value was 0.735 (95% CI: 0.342, 1.0) (Table 2).

The vast majority (84.8%) of respondents preferred to have a provider of the same gender for their pelvic examinations, while about three-in-five (59.3%) preferred to self-sample for their HPV DNA test. All said that they would test with self-sampling method again and recommend self-sampling to a friend (Table 3). For each of the five measures of acceptability of self-sampling versus clinician-sampling, self-sampling was significantly more favorable than was physician collection (all p-values were less than 0.05). The smallest difference was in how well cared for the participants felt (mean of 3.57 for self-sampled versus 3.46 for clinician-sampled), while the largest difference was in whether the test caused genital pain (mean of 3.42 for self-sampling versus 2.85 for clinician-collected) (Table 4).

**Table 1:** Demographic and Clinical Characteristics Women in Rural Mysore, India (N=120)

Characteristics	n (%) or median (IQR)
Demographic Characteristics	
Age (years)	39 [32-45]
Monthly income (INR)	5000 [3000-10,000]
Education	
None	29 (24.2)
Primary (1-4)	14 (11.7)
Middle (5-7)	25(20.8)
Secondary or above (8 or above)	50 (41.7)
Religion	
Hindu	113 (94.2)
Muslim	6 (5.0)
Marital status	
Married	105 (87.5)
Widowed	14 (11.7)
Ever screened for cervical cancer	
No	64 (53.8)
Yes	4 (3.4)
Don’t Know	51 (42.9)

**Table 2a:** Screening Results among Women Attending Mobile Cervical Cancer Screening in Mysore, India. (N=119)

Measure	n (%)
Self-Sampled cervicovaginal HPV DNA	
Positive	12 (10.1)
Negative	107 (89.9)
Clinician-sampled cervicovaginal HPV DNA	
Positive	15 (12.6)
Negative	104 (87.4)

**Table 2b:** Accuracy and Test Concordance Comparing Self- and Clinician-Sampled HPV DNA among Women Attending Mobile Cervical Cancer Screening in Rural Mysore, India (N=119)

	Estimate	95% CI
Sensitivity	66.7	42.8, 90.6
Specificity	98.1	95.5, 100.0
Positive predictive value	83.3	62.2, 100.0
Negative predictive value	95.3	91.3, 99.3
κ-statistic	73.5	34.2, 100.0
Total agreement	94.1	88.1, 97.3
HPV positive agreement	66.7	41.5, 85.0

**Table 3:** Cervical Cancer Screening Examination Preferences (n=118)

Measure	n (%)
Gender preference for provider	
Male	1 (0.9)
Female	100 (84.8)
No preference	17 (14.4)
Collection type preference for HPV DNA test	
Self-sampling	70 (59.3)
Clinician-sampling	33 (28.0)
No preference	15 (12.7)
Test with self-sampling again (n=117)	
No	0 (0%)
Yes	117 (100%)
Recommend self-sampling to a friend	
No	0 (0%)
Yes	118 (100%)

*Range from 1 to 5, 1, least favorable; 5, most favorable

**Table 4:** Acceptability of Self-Sampling Compared to Clinician-Sampling for HPV DNA Testing (N=118)

Acceptability Measure	Self-sampling Mean (SD)	Physician-sampling Mean(SD)	p-value
How well cared for did you feel?	3.57 (0.70)	3.46 (0.63)	0.005
How well was your privacy handled during the test?	3.61 (0.66)	3.47 (0.59)	<0.001
Did you feel embarrassed?	3.47 (0.68)	3.25 (0.63)	<0.001
Did the test cause you any genital discomfort?	3.47 (0.64)	3.00 (0.94)	<0.001
Did the test cause you any genital pain?	3.42 (0.67)	2.85 (1.06)	<0.001

*Range from 1 to 5, 1, least favorable; 5, most favorable

## Discussion

This study was designed to inform the feasibility and acceptability for conducting community based cervical cancer screening programs using mobile clinics in rural communities in India. The study has reiterated that self-sampling of vaginal samples was feasible and acceptable to women living in rural India. In fact, they preferred self-sampling and were comfortable with the process. By using self-collected samples to rule out HPV infection, it is possible to reduce missed opportunities for screening in rural India. 

Additional considerations for the feasibility of large-scale HPV self-sampling campaigns concern accuracy of results of the self-sampling process. This study found comparable performance and accuracy of self-sampling for detection of high-risk HPV DNA. There have been several studies comparing HPV screening results from self-collected and clinician-collected specimens in low- and middle-income countries with all demonstrating high levels of agreement (Bhatla et al., 2009; Quincy et al., 2012; Verma and Khanna, 2013; Nilyanimit et al., 2014; Rosenbaum et al., 2014; Adamson et al., 2015; Ma’som et al., 2016). A review and meta-analysis of 21 studies did not detect statistically significant difference in sensitivity of self-collected specimens compared to clinician-collected specimens. The authors suggested a pooled analysis of sensitivity data to detect whether sampling methods yielded statistically different sensitivities. The majority of studies in the review reported similar specificity for both sampling methods (Snijders et al., 2013). Clinically, the data suggested that self-sampling of HPV specimens was a viable alternative to clinician-sampling. 

*Interpretation in light of other evidence*

Consistent with previous studies comparing self-sampled to clinician-collected specimens, we found that self-sampled HPV specimens have a great potential to be used in community-based, screening programs. A review of nine studies found that participation in a cervical cancer screening program increased from 8.7% to 39% when self-sampling was offered as a screening option (Snijders et al., 2013). Overall, current data suggest that a self-sampling option can increase participation in cervical cancer programs in India, and other low- and middle-income countries. While there have been reports of their successful use in Africa, Latin America and in developed countries, there is limited data of their use in India (Lazcano-Ponce et al., 2011; Rees et al., 2018).

This is also one of the few studies that examined the usefulness of self-sampled swabs for cervical cancer screening in rural India (A Peedicayil et al., 2016; Adsul et al., 2019). Furthermore, the laboratory assessments of both modes of sample collection revealed high concordance when comparing specimens collected by self-sampling and clinician-collected swabs. Thus, self-sampled swabs were acceptable to a substantial group of rural women and were reliable in terms of the detection of HPV DNA making them one of the preferred methods to be used in population based surveillance of reproductive cancers such as cervical cancer.

*Limitations*

This study should be considered in light of its limitations. First, since this was a pilot study to evaluate the interest and acceptability of using self-sampling before rolling out the large screening program, the sample size was small. It is likely that women may have self-selected to participate in the study and hence the findings may not be generalizable. Second, it is possible that there might be information bias as the acceptability information may be influenced by the fact that the women were asked to respond to the survey by an interviewer. Despite these limitations, this was one of the first studies to examine the feasibility and acceptability of using self-sampling techniques in a community based setting in a rural area of India. We used standardized tests to screen for HPV DNA and all study staff were trained to administer the surveys in a non-judgmental manner. 

Based on the findings from this study, PHRII has used self-collected swabs as one of the main methods for specimen collection in all their subsequent community studies of cervical cancer screening in rural Mysore, India. This study demonstrated that self-sampled swabs were preferred by a large number of women, especially when the instructions on collection were provided in a user friendly, non-judgmental manner.

In conclusion, this study demonstrates that HPV self-sampling for cervical cancer screening is feasible and acceptable in a community setting among South Indian rural women. Concordance between self-sampling and clinician-sampling was adequate for screening in community settings raising the possibility that large scale screening programs could reduce the burden of cervical cancer cases and deaths in India. 

## Author Contribution Statement

Dr. Madhivanan had full access to all data in the study and takes responsibility for the integrity of the data and accuracy of the data analysis. All authors accept responsibility for the paper as published. Study concept and design: Nishimura, Krupp, Ravi, Jaykrishna, Madhivanan, Srinivas, Arun. Acquisition of data: Nishimura, Ravi, Krupp, Jaykrishna, Srinivas, Arun. Analysis and interpretation of data: Nishimura, Pope, Coudray, Madhivanan, Jaykrishna, Krupp. Drafting of the manuscript: Madhivanan, Pope, Krupp, Ravi, Coudray, Nishimura. Critical revision of the manuscript for important intellectual content: All authors.
